# IL-1β promotes Th17 differentiation by inducing alternative splicing of FOXP3

**DOI:** 10.1038/srep14674

**Published:** 2015-10-06

**Authors:** Reiner K. W. Mailer, Anne-Laure Joly, Sang Liu, Szabolcs Elias, Jesper Tegner, John Andersson

**Affiliations:** 1 Translational Immunology Unit, Department of Medicine Solna, Karolinska Institutet, Stockholm, Sweden; 2 Unit of Computational Medicine, Department of Medicine Solna, Karolinska Institutet, Stockholm, Sweden

## Abstract

CD4^+^FOXP3^+^ regulatory T (Treg) cells are essential for maintaining immunological self-tolerance. Treg cell development and function depend on the transcription factor FOXP3, which is present in several distinct isoforms due to alternative splicing. Despite the importance of FOXP3 in the proper maintenance of Treg cells, the regulation and functional consequences of FOXP3 isoform expression remains poorly understood. Here, we show that in human Treg cells IL-1β promotes excision of *FOXP3* exon 7. FOXP3 is not only expressed by Treg cells but is also transiently expressed when naïve T cells differentiate into Th17 cells. Forced splicing of FOXP3 into FOXP3Δ2Δ7 strongly favored Th17 differentiation *in vitro*. We also found that patients with Crohn’s disease express increased levels of *FOXP3* transcripts lacking exon 7, which correlate with disease severity and IL-17 production. Our results demonstrate that alternative splicing of FOXP3 modulates T cell differentiation. These results highlight the importance of characterizing FOXP3 expression on an isoform basis and suggest that immune responses may be manipulated by modulating the expression of FOXP3 isoforms, which has broad implications for the treatment of autoimmune diseases.

CD4^+^FOXP3^+^ regulatory T (Treg) cells suppress immune activation in a dominant manner and are essential for maintenance of immunological tolerance^1^. Treg cells depend on the forkhead/winged-helix transcription factor FOXP3 to be able to exert their function^2^,^3^,^4^. The importance of FOXP3 for efficient regulation of the immune system is best illustrated by the development of lethal lymphoproliferative disease in both mice and humans with genetic deficiencies in FOXP3, known respectively as scurfy and immune dysregulation, polyendocrinopathy, enteropathy, X-linked (IPEX) syndrome^5^,^6^,^7^. Deficiency in Treg cell function has also been suggested as an underlying cause for disease conditions ranging from autoimmune diseases to infectious diseases^8^.

The *FOXP3* gene encodes a transcription factor that contains three functional domains including a proline-rich N-terminal domain encoded by exons 2–4, a zinc finger and leucine zipper domain encoded by exons 5–7, and a fork-head domain encoded by exons 9–11^9^,^10^,^11^,^12^. The proline-rich N-terminal domain of FOXP3 enables the protein to interact with transcriptional repressors and activators, which consequently alter gene expression and regulate the suppressive ability of Treg cells^13^. The zinc finger and leucine zipper domain of FOXP3 is necessary and sufficient to undergo homo-oligomerization and hetero-association with other transcription factors, such as FOXP1^9^,^11^,^14^,^15^. Lastly, FOXP3 has a high specificity for gene regulation, conferred by the C terminal fork-head domain, which mediates DNA binding of FOXP3^12^,^16^.

Alternative splicing is a strictly regulated process wherein particular exons of a pre-mRNA are either included in or excluded from the mature mRNA. Alternative splicing consequently allows a single gene to give rise to multiple proteins that can have different or even opposing functions. The two most abundant FOXP3 isoforms, full-length FOXP3 (FOXP3fl) and FOXP3 lacking exon 2 (FOXP3Δ2), confer suppressive ability to Treg cells^17^,^18^. In contrast, FOXP3 lacking exons 2 and 7 (FOXP3Δ2Δ7) has been reported to inhibit other FOXP3 isoforms in a dominant negative manner^19^. A recent study has also demonstrated that exon 7 of FOXP3 is required for proper Treg cell function, as two different point mutations located near the intron 7 splice donor site result in excision of FOXP3 exon 7 and IPEX syndrome^20^. Despite the importance of FOXP3 in Treg cells, the regulation and functional consequences of FOXP3 isoform expression remains poorly understood.

## Results

### Alternative splicing of FOXP3 in patients suffering from Crohn’s disease

Based on the suggested counter-suppressive activity of FOXP3Δ2Δ7, we hypothesized that lack of FOXP3 exon 7 could contribute to the pathogenesis of chronic inflammatory diseases. To determine the role of exon 7 loss in the pathogenesis of chronic inflammatory disease, we examined the expression of *FOXP3* splice variants in patients suffering from Crohn’s disease by real time PCR using primers targeting exon/exon boundaries of exon 2 and exon 7. This allowed us to distinguish between *FOXP3* mRNA containing exon 2 (*FOXP3ex1/2*; i.e. *FOXP3fl* and *FOXP3Δ7*), *FOXP3* mRNA lacking exon 2 (*FOXP3ex1/3*; i.e. *FOXP3Δ2* and *FOXP3Δ2Δ7*), and *FOXP3* mRNA lacking exon 7 (*FOXP3ex6/8*; i.e. *FOXP3Δ7* and *FOXP3Δ2Δ7*). According to this nomenclature the primer sets can specifically amplify mRNA molecules when the listed exons are adjacent to each other. To adequately compare the abundance of the different splice variants, we determined and compensated for the efficiency of the different primer sets (Supplementary Fig. 1). The total amount of *FOXP3* mRNA was calculated as the sum of *FOXP3ex1/2* and *FOXP3ex1/3* mRNA, which allowed us to determine the relative proportion of a specific splicing event.

**Figure 1:**
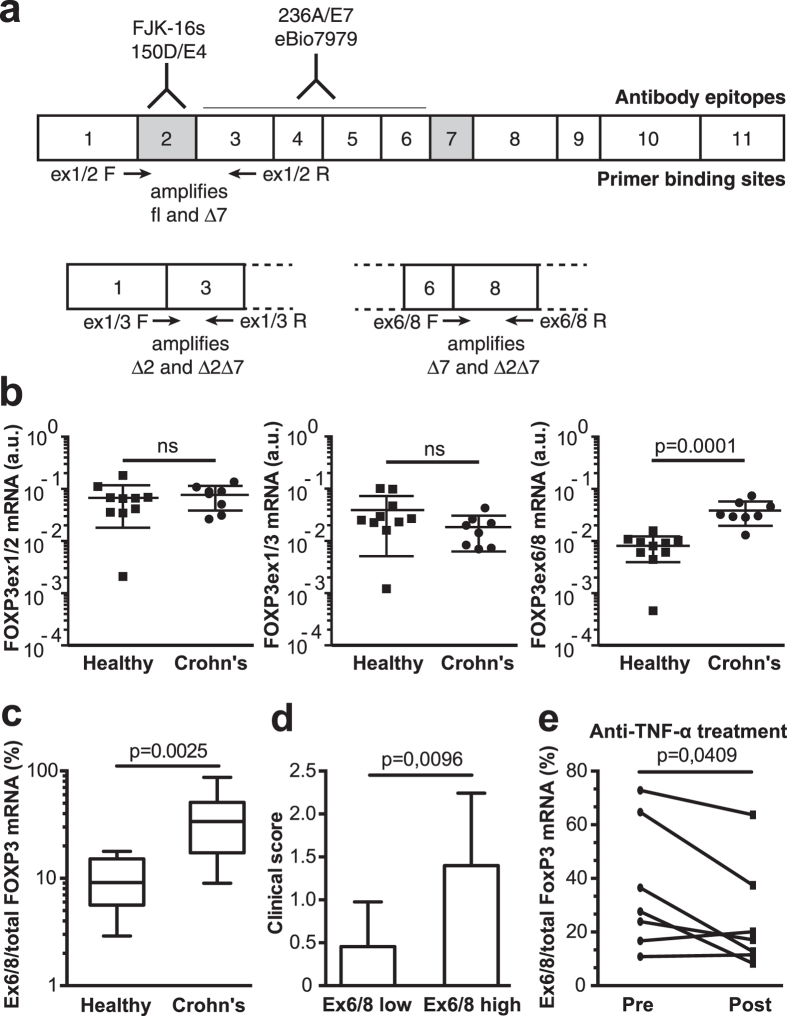
Increased alternative splicing of *FOXP3* exon 7 in Crohn’s disease. (**a**) Schematic overview of FOXP3 depicting alternatively spliced exons, epitopes of antibody clones and binding sites for primers used for detection of each splice variant. (**b**) Real-time PCR quantification of *FOXP3* transcripts expressing exon 2 (*FOXP3ex1/2*), lacking exon 2 (*FOXPex1/3*), and lacking exon 7 (*FOXP3ex6/8*) in PBMCs obtained from Crohn’s disease patients (*n *= 8) or healthy donors (*n *= 10). (**c**) Percentage of *FOXP3ex6/8* transcripts in relation to total *FOXP3* transcripts in PBMCs (*n *= 11) of Crohn’s disease patients and healthy donors (*n *= 10). (**d**) Crohn’s disease colon biopsies (*n *= 29) stratified into 50% lowest and highest *FOXP3ex6/8* expression samples plotted versus clinical score. (**e**) Percentage of *FOXP3ex6/8* transcripts in relation to total *FOXP3* in intestinal biopsies obtained from Crohn’s disease patients (*n *= 7) before and after successful anti-TNF-α treatment. (**b–e**) Data represent one pooled experiment with *n* biological replicates and are presented as (**b,d,e**) mean ± SD, (**c**) median ± IQR. P < 0.05 was considered significant ((**b,d**) two-tailed unpaired Student’s *t* test, (**c**) *Kruskal*-*Wallis ANOVA* and *Dunn’s* post hoc test, (**e**) two-tailed paired Student’s *t* test).

The total amount of *FOXP3* mRNA was 0.11 ± 0.026 arbitrary units (mean ± SEM, n = 11) in Crohn’s disease patients, which did not significantly differ (two-tailed unpaired Student’s *t* test) from the levels found, 0.095 ± 0.018 (mean ± SEM, n = 10), in healthy donors. We found that the absolute amount (Fig. 1b) and relative abundance of *FOXP3ex6/8* mRNA (Fig. 1c), but not *FOXP3ex1/2* or *FOXP3ex1/3* was greater in Crohn’s disease patients than in healthy donors. This increased expression of *FOXP3ex6/8* mRNA correlated with increased disease severity (Fig. 1d). Furthermore, patients with Crohn’s disease displayed slightly decreased proportions of *FOXP3ex6/8* when successfully treated with anti-TNF-α antibodies (Fig. 1e).

### IL-1β promotes excision of FOXP3 exon 7

**Figure 2:**
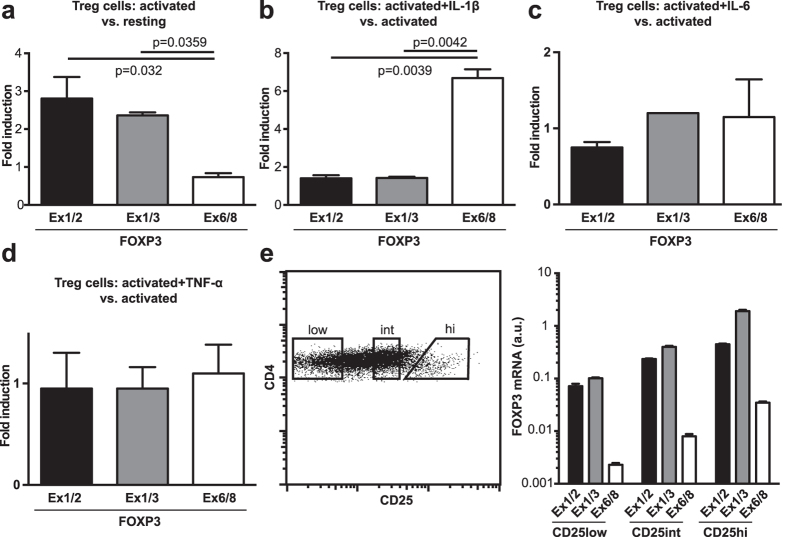
Activation and IL-1β regulate alternative splicing of *FOXP3.* Quantitative PCR was used to analyze: (**a**) fold induction of *FOXP3* transcripts in CD4^+^CD25^hi^CD127^low^ cells activated with plate bound α-CD3 and soluble α-CD28 and IL-2 for 18 hours relative to freshly isolated cells. (**b-d**) Fold induction of *FOXP3* transcripts in CD4^+^CD25^hi^CD127^low^ cells activated as above in the presence of 10 ng/ml IL-1β, IL-6 or TNF-α relative to cells activated without cytokines. (**e**) *FOXP3ex1/2* (dark gray), *FOXP3ex1/3* (gray), and *FOXP3ex6/8* (white) transcripts in CD4^+^ T cells sorted for low, intermediate (int), and high (hi) expression of CD25. (**a,b**) Data are representative of four independent experiments and presented as mean ± SD. P < 0.05 was considered significant (two-tailed unpaired Student’s *t* test). (**c**) Data (*n *= 3 technical replicates) are presented as mean ± SD.

Having determined that patients suffering from Crohn’s disease had an increased frequency of exon 7 splicing in *FOXP3* mRNA, we went on to identify factors that modulate the alternative splicing of *FOXP3* in human Treg cells. To examine whether activation of Treg cells altered the balance of FOXP3 isoforms we compared the expression of *FOXP3* transcripts between freshly isolated Treg cells and Treg cells activated with anti-CD3 and IL-2. We observed that *FOXP3ex1/2* and *FOXP3ex1/3* mRNA, but not *FOXP3ex6/8* mRNA, were upregulated upon activation (Fig. 2a). We reasoned that signals promoting immune responses might alter the splicing of *FOXP3* mRNA molecules to modulate their proinflammatory actions. Therefore, we addressed whether proinflammatory cytokines could modify the expression of *FOXP3* splice variants in Treg cells. The expression of amount of total *FOXP3, FOXP3ex1/2* and *FOXP3ex1/3* mRNA were unchanged in response to TCR stimulation regardless of the presence of IL-1β, IL-6 or TNF-α (Fig. 2b–d, data not shown). Interestingly, although *FOXP3ex6/8* mRNA was unchanged in response to TCR stimulation combined with IL-6 or TNF-α (Fig. 2c–d), it was increased in response to TCR stimulation supplemented with IL-1β (Fig. 2b).

It is also possible that subsets of Treg cells differ in their expression of *FOXP3* splice variants. To address this possibility we isolated CD4^+^ T cells based on their expression of CD25, a component of the high affinity receptor for IL-2, and analyzed the expression of *FOXP3* splice variants using qPCR. We found that the total amount of *FOXP3* mRNA correlated with the degree of CD25 expression, but no difference in *FOXP3* splice variant distribution was apparent (Fig. 2e). Taken together, these results indicate that environmental cues regulate alternative splicing of *FOXP3* and consequently the function of FOXP3.

### Increased splicing frequency of FOXP3 exon 7 promotes Th17 differentiation

**Figure 3:**
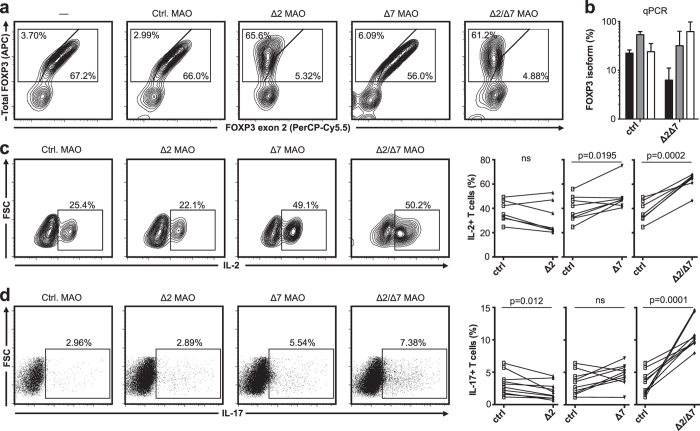
FOXP3Δ2Δ7 promote IL-17A production in naïve T cells. (**a**) Density plots of FOXP3 expression (total and exon 2) in enriched CD25^+^CD4^+^ T cells (*n *= 10), that had been transfected with control MAO (control), or with splice-redirecting MAO specific for *FOXP3* exon 2 (MAO Δ2) and/or MAO specific for *FOXP3* exon 7 (MAO Δ7). (**b**) Real-time PCR quantification of *FOXP3ex1/2* (black), *FOXP3ex1/3* (gray) and *FOXP3ex6/8* (white) transcripts of naïve T cells transfected with control MAO or MAO Δ2 and MAO Δ7 (*n *= 10). Expression was normalized to *HPRT-1* and transcription ratio was calculated relative to total *FOXP3.* (**c,d**) MAO transfected CD4^+^ Naïve T cells were differentiated towards the Th17 lineage for 5 days and (**c**) IL-2 (*n *= 7) and (**d**) IL-17A (*n *= 10) cytokine expression were assessed by flow cytometry. Data are presented as mean ± SD, P<0.05 was considered significant,two-tailed paired Student’s *t* test.

Previous studies have demonstrated that FOXP3Δ2Δ7 is incapable of conferring suppressive ability to T cells^19^. However, FOXP3 is not only expressed by Treg cells but it is also transiently expressed during Th17 differentiation. Because IL-1β promotes both alternative splicing of FOXP3 and Th17 differentiation, we next assessed the ability of FOXP3 isoforms to modulate Th17 differentiation. We purposely did not use overexpression of FOXP3 isoforms as T cell activation induces relatively high levels of endogenous FOXP3fl and FOXP3Δ2. Instead, we altered the splicing pattern of FOXP3 using morpholino antisense oligonucleotides (MAO) that prevent splice-directing small nuclear ribonucleoproteins from binding the exon/exon boundaries of *FOXP3* pre-mRNA^21^. We demonstrated that it is possible to remove *FOXP3* exon 2 in Treg cells with great efficiency (Fig. 3a) but the MAO targeting exon 7 was less efficient and could only partially remove *FOXP3* exon 7 in Treg cells as determined by qPCR (data not shown). On the other hand MAO targeting efficiently removed *FOXP3* exon 7 in naïve T cells upon differentiating into Th17 cells, presumably because MAO transfection precedes the induction of *FOXP3* mRNA expression and the total amount of *FOXP3* mRNA is much lower in these cells (Fig 3b). MAO-mediated splice shifting was observed as early as 24 hours post-transfection and persisted for at least 1 week (data not shown). Importantly, in these cells we found that expression of IL-2 (Fig. 3c) and IL-17A (Fig. 3d), but not IFN-γ (data not shown), is modulated by alternative splicing of *FOXP3*. Indeed, the combined removal of exon 2 and exon 7 mediated by MAO treatment strongly enhanced expression of IL-2 and IL-17A (Fig. 3c,d).

### Increased splicing frequency of FOXP3 exon 7 correlate with IL-17A expression *in vivo*

**Figure 4:**
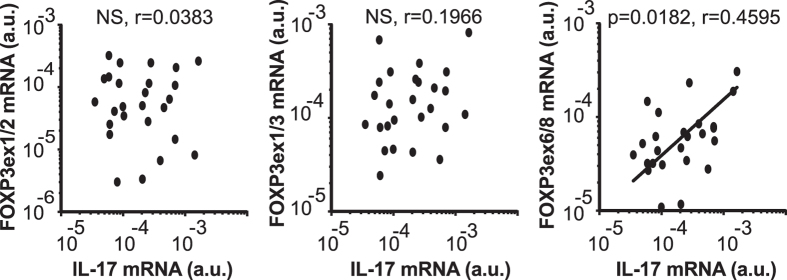
Alternative splicing of *FOXP3* exon 7 correlates with IL-17A expression. Quantitative PCR was used to measure *FOXP3* and *IL-17A* transcript levels in colon biopsies from patients suffering from Crohn’s disease and normalized to *GAPDH* expression (*n *= 26). Data are presented as mean of *n *= 3 technical replicates. P < 0.05 was considered significant, Spearman rank correlation test; *r *= correlation coefficient).

Based on research with murine cells indicating that exon 2 of Foxp3 binds to and antagonizes ROR-γt^22^,^23^, it has been suggested that FOXP3Δ2 may be linked to increased levels of IL-17 production in humans. However, a recent study with patients suffering from inflammatory bowel disease could not verify such an association^24^. Here, we analyzed biopsies obtained from Crohn’s disease patients and determined the levels of *FOXP3* mRNA splice variants and cytokine mRNA expression. In accordance with previous studies we found no correlation between *FOXP3ex1/3* and IL-17A expression. However, we observed a significant positive correlation between the levels of *FOXP3ex6/8* mRNA and *IL-17A* mRNA (Fig. 4). Furthermore, no correlations were evident between FOXP3 splice variants and IFN-γ (data not shown), suggesting that alternative splicing of *FOXP3* specifically affects Th17 differentiation. Altogether, these results suggest that FOXP3Δ2Δ7 favors the differentiation of naïve T cells towards Th17 cells in the setting of Crohn’s disease.

## Discussion

In this study, we demonstrate that patients suffering from Crohn’s disease display an irregular pattern of *FOXP3* splicing with an increased proportion of *FOXP3* transcripts lacking exon 7. We also found that the excision of *FOXP3* exon 7 is controlled by the proinflammatory cytokine IL-1β and show that this *FOXP3* splicing event promotes the differentiation of naïve T cells into Th17 cells.

Alternative splicing is the process by which exons of RNA are reconnected in multiple ways during RNA splicing^25^. Its is a major contributor to transcriptome and proteome diversity and recent studies indicate that up to 95% of human pre-mRNAs that contain more than one exon are processed to yield multiple mRNAs^26^. There are now several prominent examples of regulation of immune responses through alternative splicing^27^. For example, MyD88 an adaptor protein involved in Toll like receptor signaling, is found in two different isoforms with opposing function, where the MyD88_L_ isoform activates the innate immune responses and the MyD88_S_ isoform that lacks exon 2 inhibits immune responses^28^,^29^. In a similar manner activated T cells produce a soluble form of the common γ-chain, where exon 6 have been excised from the mature mRNA transcript, resulting in a new 9-amino-acid epitope followed by a stop codon. This soluble form of the common γ-chain inhibits cytokine signaling and consequently opposes the function of the full-length common γ-chain^30^,^31^. These and other examples of isoforms having opposing functions may illustrate that alternative splicing is a fast approach for turning off a biological response, as a dominant negative form can interfere with the function of already translated proteins that otherwise would continue to function.

Many questions remain regarding the function of FOXP3Δ2Δ7, however it is becoming increasingly clear that this isoform is unable to confer suppressive ability to Treg cells. Post-translational modifications, such as acetylation of the lysine-rich region in exon 7, have been proposed to stabilize FOXP3. However, when analyzing MAO-treated Treg cells treated with the proteasome inhibitor MG132 it appeared that FOXP3Δ2Δ7 is as stable as the other FOXP3 isoforms (data not shown). FOXP3Δ2Δ7 does however maintain the ability to bind DNA (data not shown) and presumably acts in a dominant negative manner by displacing FOXP3fl and FOXP3Δ2. The arguably most important aspect of this study is that we were able to identify that IL-1β promotes the excision of exon 7 of FOXP3. This gives us a valuable tool when we in the future want to study the molecular mechanisms of FOXP3 splicing. It also illustrates a novel mechanism as how a proinflammatory environment can tune the function of Treg cells through alternative splicing of FOXP3. A previous study has shown increased mRNA level of *FOXP3Δ2Δ7* in patients suffering from rheumatoid arthritis^37^. Thus it would appear that altered splicing of FOXP3 mRNA is shared among several distinct inflammatory disorders, which is expected considering that IL-1β expression is a prominent feature during inflammation. However before we are able to fully elucidate the function of different FOXP3 isoforms, we need a new set of tools that will allow us to quantify FOXP3 isoforms on a protein level. While this additional layer of analysis could initially prove cumbersome, it may also resolve controversies where expression of FOXP3 does not correlate with an expected anti-inflammatory phenotype.

The intestinal immune system has to provide an effective immune response against pathogenic bacteria while maintaining tolerance towards food and commensal flora^32^,^33^ and T helper cells control the type of immune response that is mounted. Therefore it is not surprising that inflammatory bowel diseases are characterized by the excessive activation of certain T helper subsets such as Th1 and Th17. Recent genome-wide association studies have demonstrated that a number of genes involved in Th17 differentiation/function (IL23R, IL12B, JAK2, STAT3, CCR6 and TNFSF15) are associated with susceptibility to Crohn’s disease^34^,^35^,^36^,^37^,^38^. Treg cells are found in increased numbers in the intestinal mucosa of patients suffering from Crohn’s disease^39^, but appear unable to break the chronic inflammatory state. This could in part be due to increased expression of FOXP3∆2∆7, which is unable to confer a suppressive phenotype to Treg cell *in vitro*^19^. While several studies demonstrated that Treg cells from inflammatory bowel disease patients are functional^40^,^41^,^42^, there are also suggestions that these cells can alter their lineage commitment in response to the extracellular environment. In fact, patients with inflammatory bowel disease exhibit higher prevalence of circulating IL-17 and FOPX3 double positive CD4+ T cells^43^. In this study we did not assess the impact of FOXP3∆2∆7 on Treg cell plasticity, however, our study could potentially provide a molecular explanation for such Treg cell plasticity. Previous studies have demonstrated a key role of IL-1β as Helios-FOXP3^+^ Treg cells downregulate their suppressive functions in response to IL-1β^44^. In addition, Treg cells exposed to both IL-1β and IL-2 differentiate into proinflammatory Th17 cells^45^. It is undoubtedly a field that merits further investigations in order to establish a functional link between inflammation and autoimmunity.

TGF-β regulates the differentiation of both Treg cells and Th17 cells by inducing transient expression of both FOXP3 and RORγt. Foxp3 directly binds to and antagonizes ROR-γt^22^,^23^. The binding and inhibition of ROR-γt were to a large extent dependent on exon 2 of Foxp3. In addition, Zhou *et al.* also noted that knockdown of Foxp3 during Th17 differentiation resulted in an increase in Th17 cells^23^. Here, we determined that preferential expression of the FOXP3∆2∆7 isoform facilitated Th17 differentiation, which agrees with the latter finding, as FOXP3∆2∆7 inhibits the function of FOXP3fl and FOXP3∆2 in a dominant-negative manner. Our finding that FOXP3∆2∆7 facilitates Th17 differentiation is further supported by the correlation between FOXP3∆2∆7 expression and IL-17 (but not IFN-γ) expression in patients suffering from Crohn’s disease. By contrast, a recent study by Lord *et al.* suggests that no such correlation exists between FOXP3∆2 expression and IL-17 production in patients suffering from inflammatory bowel disease^24^. Thus, FOXP3 isoforms may regulate Th17 differentiation in two distinct ways: (a) by FOXP3fl directly binding to and antagonizing ROR-γt or (b) by general inhibition of FOXP3 function through upregulation of the dominant negative isoform FOXP3∆2∆7. The ability of IL-1β to promote Th17 differentiation is only partially conserved across species. Chung *et al.* have demonstrated that IL-1 receptor 1 expression in T cells, which is induced by IL-6 signaling, is necessary for early Th17 cell differentiation *in vivo* in mice^46^. Taking into account that alternative splicing of FOXP3 does not occur in mice, the latter study strongly supports the hypothesis that IL-1β modulates Th17 differentiation in more ways than simply inducing alternative splicing of *FOXP3*.

In summary, our study highlights the importance of characterizing FOXP3 expression on an isoform basis, as different FOXP3 isoforms are differentially regulated and exhibit distinct functional characteristics. Splicing of FOXP3 may be an important physiological regulator of Treg cell function and T-cell lineage commitment. Importantly, we found that the proinflammatory cytokine IL-1β promotes excision of exon 7 of FOXP3 by alternative splicing resulting in increased Th17 polarization. As such, our results shed new light on the mechanisms underlying chronic inflammatory diseases, such as Crohn’s disease, providing new pathways that could be targeted for treatment of these diseases.

## Material and Methods

### Patient Samples

Peripheral blood mononuclear cells (PBMCs) and biopsies from affected areas of the rectum and sigmoid colon were obtained from patients with Crohn’s disease. Disease activity was graded according to the simplified endoscopic activity score for Crohn’s disease as previously described^47^. A subset of patients was treated with the anti-TNF-α antibodies, infliximab or adalimumab (Remicade® or Humira®, respectively). Patients’ response to treatment was assessed using the Harvey–Bradshaw Index^48^. Patients with a clinical index activity decrease by ≥3 points were regarded as responders. The choice of treatment for individual patient was based on clinical evaluation without any intervention by the study. Anti-TNF-α treatment was administered either as infusions of 5 mg/kg infliximab at weeks 0, 2 and 6 or as subcutaneous injections of 80 mg adalimumab at week 0 followed by 40 mg every other week.

### Ethical considerations

The study was approved by the Ethical Committee of Northern Stockholm and written informed consent was obtained from all participants. All experiments were performed in accordance with relevant guidelines and regulations.

### Antibodies

The antibody clones used throughout this study are listed in Supplementary Table I.

### Isolation of T cells

PBMCs were isolated from buffy coats using Ficoll-Paque Plus gradient centrifugation (GE Healthcare). CD4^+^ T cells were enriched from PBMCs using positive selection on an AutoMACS Separator with human CD4 micro-beads (Miltenyi Biotec). The enriched CD4^+^ T cells were then stained with antibodies recognizing CD4, CD25, CD127 (Supplementary Table I). CD4^+^CD25^hi^CD127^low^ Treg cells were sorted using a FACSJazz instrument (BD Biosciences). Untouched naive CD4^+^ T cells were isolated from PBMCs by depleting non-T helper cells and memory CD4^+^ T cells using naïve CD4+ T cell Isolation Kit II according to the manufacturer’s instructions (Miltenyi Biotec). The enriched CD4^+^ T cells were then stained with antibodies recognizing CD4, CD25, CD45RA and CD62L (Supplementary Table I). Highly purified naïve T cell populations (>95%) were obtained by sorting for CD4^+^CD25^−^CD45RA^+^CD62L^−^ cells using a FACSJazz instrument.

### Cell culture and Th17 differentiation *in vitro*

T cells were activated and grown in X-VIVO 15 Medium supplemented with 1% Penicillin-Streptomycin (both from Lonza), 5 μg/ml plate-bound α-CD3, 1 μg/ml soluble α-CD28 (both from Biolegend), 300 U/ml IL-2 (Preprotech) and 6% CO_2_ at 37 °C. Naïve T cells were differentiated towards the Th17 lineage by culturing them with 10 ng/ml TGF-β, 10 ng/ml IL-1β, 25 ng/ml IL-6 and 10 ng/ml IL-23 (all from Peprotech) for 6 days. Four hours prior to harvesting, cells were restimulated with 50 ng/ml PMA (Sigma) and 1 μg/ml ionomycin (Life Technologies) and treated with GolgiBlocker (BD Biosciences).

### Splice-shifting

Enhanced FOXP3 exon splicing was achieved using Fluorescein-labelled Morpholino Antisense Oligonucleotides (MAO) with the following sequences: 5′-TGCCCATTCACCGTCCATACCTGGT-3′ for *FOXP3*ex1/3, 5′-AGCTGTGAAATGGCACAAACATGAG-3′ for *FOXP3*ex6/8 and 5′-CCTCTTACCTCAGTTACAATTTATA-3′ as a control (GeneTools). Prior to activation, T cells were transfected with 15 μM MAO using the P3 Primary Cell Nucleofector Kit and a Nucleotransfector device (Lonza) according to the manufacturer’s instructions.

### FACS

Single-cell suspensions were stained with LIVE/DEAD Fixable Dead Cell Stain Kit (Life Technologies) to identify dead cells. Intracellular staining was performed using eBioscience’s FOXP3 staining kit according to the manufacturer’s instructions. Data was acquired on a LSRFortessa flow cytometer (BD Biosciences) and analyzed with FlowJo Version 7.6.4 software for Mac (TreeStar).

### Quantitative PCR

Total RNA from T cells was isolated with Trizol (Life Technologies) and cDNA was generated using Vilo cDNA Synthesis Kit (Life Technologies). Amplification was performed with iQ SYBR Green Supermix (Bio-Rad) with the following protocol: 2 min 95 °C, (15 sec 95 °C, 45 sec 58 °C, 30 sec 68 °C) × 39. Gene specific primer pairs are listed in Supplementary Table II. Threshold cycles (Ct) calculated by Bio-Rad CFX software were normalized to the expression of *GAPDH* (*ex vivo* samples) or *HPRT1* (cell culture samples). Relative amounts were calculated with [c] = 2^ΔCt^, changes in expression levels with [fold] = 2^−ΔΔCt^. Primer specificity in all samples was confirmed by single peak performances of PCR products in melt curve analysis. Expression of *FOXP3* splice variants was calculated with respect to their individual primer pair efficiency (Supplementary Fig. 1). Total *FOXP3* mRNA expression was calculated as the sum of *FOXP3ex1/2* and *FOXP3ex1/3* and splice variant percentage was calculated as (*FOXP3 variant*/*total FOXP3*)*100.

## Additional Information

**How to cite this article**: Mailer, R. K. W. *et al.* IL-1b promotes Th17 differentiation by inducing alternative splicing of FOXP3. *Sci. Rep.* **5**, 14674; doi: 10.1038/srep14674 (2015).

## Supplementary Material

Supplementary Information
