# Supplementary material for: IL-1β promotes Th17 differentiation by inducing alternative splicing of FOXP3
Source: Sci Rep. 2015 Oct 6;5:14674. doi: 10.1038/srep14674 (PMC4593960; doi:10.1038/srep14674)
Supplement: Supplementary Information [file srep14674-s1.pdf]

Supplemental information

IL-1β promotes Th17 differentiation by inducing alternative splicing of FOXP3

Authors: Reiner K.W. Mailer, Anne-Laure Joly, Sang Liu, Szabolcs Elias, Jesper Tegner and John Andersson

Supplemental information

Supplemental Figure 1. Analysis of isoform specific primer pair efficiency

Standard curves of quantitative serial dilutions of FOXP3fl or FOXP3**Δ**2**Δ**7 expression vectors (pcDNA3) against real-time PCR threshold cycles. Linear regression with slope and coefficient of determination (R_2) is shown.

Supplementary Figure 2

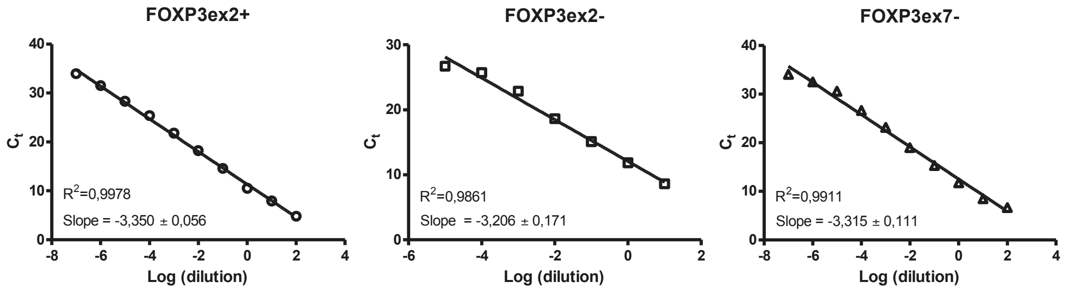

Supplemental Table I Antibody clones used

Antigen	Clone #	Provider	Application
hu CD3	OKT-3	BioLegend	Cell culture
hu CD4	RPA-T4	BD Biosciences	FACS
hu CD25	CD25-3G10	Life Technologies	FACS
hu CD28	CD28.2	BioLegend	Cell culture
hu CD45RA	HI100	BioLegend	FACS
hu CD62L	DREG-56	BioLegend	FACS
hu CD127	eBioRDR5	eBioscience	FACS
hu FOXP3 total	236A/E7	BD Biosciences	FACS
hu FOXP3 ex2+	150D/E4	eBioscience	FACS
hu IL-2	MQ1-17H12	BioLegend	FACS
hu IL-17A	BL168	BioLegend	FACS

Supplemental Table II. Primers used.

Gene	Orientation	Sequence
FOXP3 full length	sense	5'-CAGCTGCAGCTGCCCACACTG-3'
FOXP3 full length	antisense	5'-GCCTTGAGGGAGAAGACC-3'
FOXP3ex2-	sense	5'-CAGCTGCAGCTCTCAACGGTG-3'
FOXP3ex2-	antisense	5'-GCCTTGAGGGAGAAGACC-3'
FOXP3ex7-	sense	5'-GAGCAGCAGGCATCATCCG-3'
FOXP3ex7-	antisense	5'-CTGGGAATGTGCTGTTTCC-3'
IL-17A	sense	5'-GCTGCTGAGCCTGGAGG-3'
IL-17A	antisense	5'-GAGGGATATCTCTCAGGGT-3'
IFN-γ	sense	5'-GCAGCTAAAACAGGGAAGC-3'
IFN-γ	antisense	5'-AGACAGTCACAGGATATAGG-3'
GAPDH	sense	5'-GTCCACTGGCGTCTTCAC-3'
GAPDH	antisense	5'-CTTGAGGCTGTTGTCATACTTC-3'
HPRT1	sense	5'-TGACACTGGCAAAACAATGCA-3'
HPRT1	antisense	5'-GGTCCTTTTCACCAGCAAGCT-3
